# Early pregnancies among middle school students: Attribution of blame and the feelings of responsibility among teachers and parents

**DOI:** 10.3389/fpsyg.2022.987520

**Published:** 2022-10-20

**Authors:** Antony Fute, Binghai Sun, Mohamed Oubibi

**Affiliations:** Department of Psychology, College of Teacher Education, Zhejiang Normal University, Jinhua, China

**Keywords:** early pregnancy, empathy, feeling responsible, blame attribution, adolescence

## Abstract

**Introduction:**

Globally, 15% of adolescents give birth before turning 18, leading to considerable personal, social, and medical impacts on adolescents and to the general society.

**Objective:**

This study aimed at exploring and comparing three psychological attributes (i.e., empathetic concern, feelings of responsibility, and attribution of blame) between parents and teachers for the phenomena.

**Method:**

672 teachers (54% females) and 690 parents (53% female) participated in the study.

**Results:**

The results indicated a significant mean difference between parents and teachers on empathy (*t* = 5.735, *p* < 0.001), attribution of blame (*t* = 6.902, *p* < 0.001), and feelings of responsibility (*t* = 1.727, p < 0.001). Except for attribution of blame, parents’ mean scores of other variables were higher than that of teachers.

**Discussion:**

Teachers’ higher attribution of blame to pregnant adolescents and lower empathetic concern raises a prominent concern over students’ healthy environment at school.

**Conclusion:**

Understanding social feelings about responsibilities over adolescents’ general health is very essential, especially for fighting against the problem of early pregnancy.

## Introduction

Teenage pregnancy is considered a public health problem because of its considerable personal, social, and medical impacts on adolescents and the general population (Palomino Pérez et al., 2018). Globally, 15% of adolescent girls give birth before they turn 18, which is referred as early pregnancy or early childbearing (Doğan and Köse, 2022). Childbearing during teenage years may destroy girls’ healthy development into adulthood as their bodies may not be physically ready. Serious problems such as obstetric fistula, systemic infection, and eclampsia may develop in a short- or long-term (Bonner et al., 2018). The World Health Organization has ranked maternal conditions in the top five causes of death among adolescents aged 15 to 19 years, with the low- and middle-income countries contributing to 99% of it (WHO, 2020). Apart from medical complications, early pregnancies may also lead to issues of being rejected by peers or family members, stigmatized, and forced to early marriage. Generally, because of prolonged health and social problems, many pregnant adolescents, especially in developing countries, are forced to drop out of school (UNICE, 2021).

By 2018, the world had 129.2 million primary and secondary school girls out of school because of different factors, including pregnancy or childbearing (UNESCO, 2019). The reports indicate that at least 21 million adolescent girls 15–19 years old get pregnant annually in developing countries, and 10 million are unintended. Although 12 million give birth, it is approximated that 5.6 million abortions occur annually, with 3.9 million being unsafe, leading to morbidity, maternal mortality, and lasting or lifetime health problems (Ramos et al., 2017; WHO, 2020; Hakiminezhad et al., 2022). Generally, although adolescents of both sexes engage in risky behavior of early involvement with unsafe sex, girls are in the most vulnerable position of being severely affected by its outcomes compared to their boy counterparts (Nkosi et al., 2022).

**Figure 1:**
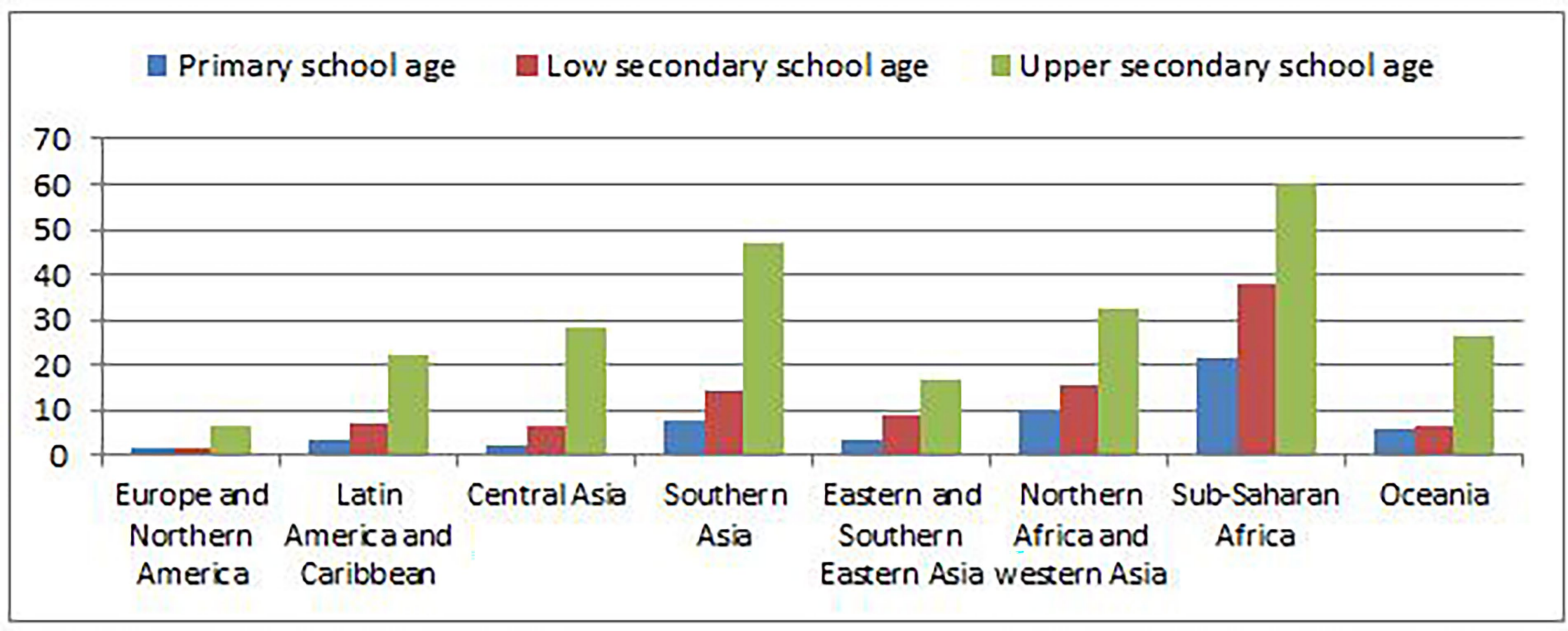
The percentage of out-of-school girl adolescents by regions.

For decades women have been socially disadvantaged because of early pregnancy, leading to many challenges, including denying their right to education. By 2019, two-thirds of the 750 million illiterate adults (500 million) globally were women, a situation that has never changed since 1976 (UNESCO, 2020). These statistics may reflect a historical record of women being vulnerable to social, political, and economic constraints. In some economically disadvantaged societies, girls are still prepared for nothing but marriage and childbearing (Misunas et al., 2021). In Sub-Saharan Africa alone, 33.6% of primary and secondary school-age girls were out of school by 2019 for different reasons, including early pregnancy. The region had the highest number of out-of-school girls worldwide, totaling 52 million, followed by Southern Asia with 45.6 million. Oceania has the least number of out-of-school girls, with 0.4 million children and adolescents (9.3%; UNESCO, 2019). Figure 1 shows the out-of-school girls by region and level of education.

Sub-Saharan African region leads by having more than 60% of secondary school-aged girls out of school. In Tanzania specifically, 3.6 million primary and secondary-aged girls are out of school because of pregnancy, while 5,500 girls drop out yearly (Fute and Wan, 2021). Although the education policy does not explicitly describe issues of the right to education among pregnant adolescents and young mothers, the government practically rusticated them from schools for several decades. Notwithstanding, as the result of internal and external forces against the exclusion and discriminative policies, in 2018, the government came up with an alternative education path for pregnant girls and young mothers (Iddy, 2021). In supporting the government’s efforts, it is essential to consider different social norms by exploring the civic responsibility toward creating and maintaining a healthy, empathetic, and stable society in which everyone’s dream is protected. Considering adolescents’ time spent at home and school, parents and teachers may play a more prominent role in changing the situation.

## Literature review

### Feelings of responsibility among teachers and parents

In the context of health, early pregnancy among middle school students is a public health problem and a concern of everyone, including parents, teachers, and students (Sciacca et al., 2021). Students’ risk behaviors like an early debut and unsafe sex are practiced at school and out of school (i.e., in homes), so the consequences of these risky behaviors are experienced (i.e., poor academic performance; Cohen-Gilbert et al., 2015). However, similar to other student behavior forms, literature has suggested that teachers and parents do not always feel automatically responsible for changing the situation (Dobson and Ringrose, 2016; Green et al., 2017).

Some teachers may feel they have full responsibility (Mura et al., 2014), while others feel being concerned about students’ behaviors outside of school would mean overstepping the school authority (Young et al., 2017). In some cases, the schools blame the victims of risky behavior or early pregnancy (Jørgensen et al., 2019). With this approach, teachers may avoid working with the victim or students’ parents toward solving the problem. We may hypothesize from this theoretical background that teachers’ feelings of responsibility toward pregnancy among middle school students are different from that of parents (H1).

### Empathy and attribution of blame

Blame attribution is the process of avoiding responsibility by blaming the victims. It is a behavior of constructing the causal explanation of the conduct by pointing at the victim as responsible for their suffering (Friedman et al., 2007). Evidence from the literature demonstrates that parents’ and teachers’ perceptions of students’ risk behaviors (i.e., sexting and early initiation of sex which may lead to early pregnancy) are very complex without a straightforward opinion on the issue (Sciacca et al., 2021). In some contexts, their perception of students’ pregnancy can be biased by their gender and students’ sex. For example, in some countries, schools have started several campaigns to prevent students’ risky behaviors that may result in pregnancy. However, most campaigns target girls and encourage them to limit sexual interaction with boys (Jørgensen et al., 2019). Targeting girls results from believing that they are provocative and blameworthy as they fail to think better about the sexual act, just like it often occurs with the victims of rape in most countries (Salter et al., 2013; Fute et al., 2022a).

In addition, teachers tend to discuss sexual matters with girls longer than they do with boys, highlighting their tendency to consider girls as the most responsible and decisive side of the sexual and other risk behaviors (Ricciardelli and Adorjan, 2019). Teachers’ and parents’ attribution to blame can be connected with individual attributes like empathy and understanding adolescents’ experiences through adopting their point of view. Empathy can also imply a concern for other people (i.e., adolescents) and their emotional experiences or feeling of distress (Donaldson et al., 2022).

A study of cyberbullying incidences among students indicated the correlation between a lower level of empathy and higher attribution of blame toward the victims (Schacter et al., 2016; Murphy et al., 2018). Empathy has also been indicated to negatively correlate with attributing blame for cases of race discrimination and rape (Muller et al., 1994). From this literature background, it can be hypothesized that (H2) higher empathy correlates with higher feelings of responsibility, (H3) parents’ and teachers’ higher empathy level correlates with lower attribution of blame, (H4) teachers’ attribution of blame is different from that of parents, and (5) females’ empathy, feelings of responsibility, and their attribution of blame is different from that of males.

### Problem of the study and objectives to achieve

Teachers and parents hold greater responsibility in shaping adolescents’ behaviors because of the time spent under their supervision, the trust adolescents put in them, and the strong connection built between them. However, although society generally condemns the increasing rate of risk behaviors and pregnancies among adolescents (i.e., students), little is known about parents’ and teachers’ empathy over the matter, attribution of blame, and their awareness of their accountability or responsibility. Thus, in addition to the ongoing government efforts like policy and legal reforms toward equal education access for all, this study explores the three psychological constructs among teachers and parents on early pregnancy among students. Empathy and feelings of responsibility are fundamental constructs for the social efforts to prevent the problem before it escalates.

## Materials and methods

### Sample size

The optimal sample size was calculated based on statistical theories (Cohen, 1988), and information from the previous studies (Norman et al., 2012; Gowda et al., 2019). A statistical software (G* Power) was used to calculate the sample size based on the assumption of 95% certain that the sample would identify a statistically significant outcome should the hypotheses be true for the population. With two tails, the “P” value for statistical significance was set at 0.05, the allocation ratio (N2/N1) at one, and small effect size (*d* = 0.02). The output parameters from the software indicated that 1,302 total sample size for each group (parents and teachers) would be scientific and ethical.

### Participants

The sample comprised 672 in-service teachers (54% women and 46% men) from 21 middle schools in Njombe region (Tanzania) and 690 parents (47% fathers and 53% mothers) whose children were studying in the sampled schools. Schools were randomly selected from the district in which according to official reports (URT, 2014; UNICEF, 2017), a number of adolescents get pregnant before they finish secondary school. Parents’ sample was randomly selected from the pool which comprised only those whose students are studying in the sampled schools. In this case, only a sample of parents whose children were studying in the 21 selected schools participated in this study. However, on the other hand, 92% of teachers in the sampled schools participated in the study. The age range of all the participants was 25–54 (Mage = 35, SDage = 2.93) for teachers and 33–62 (Mage = 42, SDage = 3.73) for parents. The majority of teachers (89.2%) had a bachelor’s level of education, while few had a master’s (2%) and diploma (8.8%). Parents had at least a primary level of education (5%,) and at most master’s level (03%). Majority of parents had secondary school education (43%), diploma education (21%), and bachelor degree (28%). The teachers’ teaching experience ranged from 2 to 32 years (Mexperience = 11.53, SDexperience = 4.21).

In order to know whether the two groups (teachers and parents) are comparable, we checked whether there are significant differences in their socio-demographic variables (age, gender, and level of education). The results (Table 1) showed insignificant mean differences between parents and teachers on age [*t*(1362) = 10.784, *p* < 0.060], level of education [*t*(1362) = 18.694, *p* < 0.089], and gender [*t*(1359) = 1.348, *p* < 0.178]. The average age for parents was 0.419 higher than the average mean score for male teachers, the average education level for parents was −1.487 less than that of teachers, and the average mean score of parents’ gender blame was −0.067 less than that of teachers.

**Table 1:** Demographic differences between teachers and parents.

	Levene’s test for equality of variances		*t*-test for equality of means					
	*F*	Sig.	*t*	df	Sig. (2-tailed)	Mean difference	95% confidence interval	
							Lower	Upper
Age	40.319	0.073	10.390	1,362	0.060	0.419	0.340	0.498
Education level	83.473	0.135	18.694	1,362	0.089	-1.487	−1.643	−1.331
Gender	0.473	0.492	1.348	1,359	0.178	−0.067	−0.163	0.030

### Procedure

Participants were recruited from 11 middle schools (teachers) and their surrounding villages (parents). A questionnaire was designed, and all the participants with the help from research assistants filled out. Teachers from the sampled schools and a few parents were invited to participate in the study, which started in March and ended in April 2022. With the help of two volunteering assistants (university students), One assistant physically visited the schools and villages to administer the survey, especially providing instructions and answering any questions teachers and parents raised. The study got approval from the first author’s university ethics committee, and all the participants were informed about the objective of the study prior to their voluntary participation. Informed consent was obtained from all the participants, and no compensation was given to participants.

### Instruments

There were four sections of the survey which were used in this study. The first section was related to participants’ demographics, whereby the participants were asked about their gender, age, education level, and teaching experience (for teachers). The second section involved a short statistical description (in one paragraph) that depicted the increasing number of adolescent pregnancies and their accompanying impacts (i.e., deaths). The third section included one standard and validated measure to assess participants’ empathy. The last section included measures of participants’ attribution of blame and feelings of responsibility.

**Empathy:** Interpersonal Reactivity Index (IRI) was used to measure participants’ empathy. The scale was developed by Davis (1983), and it has two dimensions: empathic concern (EC sub-scale) and perspective taking (PT sub-scale; Davis, 1983; Briganti et al., 2018; Fute et al., 2022b). IRI has 14 items in general, and participants were asked to rate how well these items described them. A 5-point Likert scale ranging from 1 (does not describe me well) to 5 (describes me very well) was used, and the sample of the items is: “When I see someone being taken advantage of, I feel kind of protective toward them” (EC) and “I try to look at everybody’s side of a disagreement before I make a decision” (PT). The scores of each participant for EC and PT were computed by averaging their responses across the items (α = 0.89 for EC; α = 0.78 for PT).

**Exposure to a wider reality of early pregnancy and its associated effects:** To expose a wider reality of early pregnancy and its associated impacts, participants were asked to read a paragraph that described statistically the number of adolescents who get pregnant annually. The paragraphs also described the rate of abortion (safe and unsafe), the percentage of deaths, and prolonged health problems among adolescents. The general social and economic problems that resulted from the incidences were also described (see Appendix A for full details). The idea of exposing the participants to the reality was adopted from previous studies (Wolak and Finkelhor, 2011a; Aarø et al., 2014; Sciacca et al., 2021), which used vignettes (participants read vignettes) to assess participants’ attitudes toward youths who produced and shared sexual imagery.

**Attribution of blame and feelings of responsibility:** Following the process of reading a paragraph that clearly and statistically describes the number of adolescents who get pregnant every year, all the participants were asked to express their level of agreement based on six statements which were about; (a) the attribution of blame for the targets of early pregnancy, and (b) their perceived responsibility of dealing with the problems (Oubibi et al., 2022a,b). All their answers were measured by a 5-point Likert scale which ranged from 1 (Strongly disagree) to 5 (Strongly agree). A modified version of items from Sciacca et al. (2021) was used in this study (see Appendix C), as more studies had already used the same items and showed higher reliability (Holfeld, 2014).

### Data analysis

The analysis of the data started with the coding process. SPSS 26 version was used to analyze data, whereby the descriptive statistics and correlation analysis were done for all variables, including the participants’ demographics. The second analysis involved an independent sample *t*-test for testing the hypotheses. The differences in participants’ empathy, attribution of blame, and feelings of responsibility were calculated and reported through a *t*-test table of results.

## Results

### Preliminary analysis to validate the instruments validation

Prior to hypothesis testing process, a Confirmatory Factor Analysis (CFA) was done to test the discriminant validity of the variables of our primary concern. The model fitness index was also assessed and the adopted items were validated. A series of CFA were used, including Tucker–Lewis Index (TLI), Comparative Fit Index (CFI), Expected Cross-validation Index (ECVI), Root Mean Square Error of Approximation (RMSEA), and Goodness of Fit Index (GFI). The reliability of IRI and attribution of blame and Feelings of responsibility was high (α = 0.84 and α = 0.91 respectively). All the Items from Interpersonal Reactivity Index (IRI), Attribution of blame, and the feelings of responsibility had factor loadings above 0.6, suggesting their fitness. Table 2 below shows important results from CFA analysis.

**Table 2:** Instruments validation results.

Constructs	Sub-constructs and items	Factor loading	R^2^
**Interpersonal reactivity index** (IRI; *TLI = 0.89, CFI = 0.91, GFI = 0.88, RMSEA = 0.053, ECVI = 0.96*)			
	EC1 I often have tender, concerned feelings for people less fortunate than me	0.74	0.695
	EC2 Sometimes I do not feel very sorry for others when they have problems (R)	0.82	0.901
	EC3 When I see someone being taken advantage of, I feel protective toward them.	0.79	0.784
	EC4 Other people’s misfortunes do not usually disturb me a great deal (R)	0.65	0.773
	EC5 When I see someone being treated unfairly, I sometimes do not feel very much pity for them (R)	0.78	0.796
	EC6 I would describe myself as a pretty soft-hearted person	0.70	0.861
		
	PT1 I sometimes find it difficult to see things from the “other guy’s” point of view (R)	0.69	0.793
	PT2 I try to look at everybody’s side of a disagreement before making a decision	0.83	0.837
	PT3 I sometimes try to understand my friends better by imagining how things look from their perspective.	0.71	0.856
	PT4 If I’m sure I’m right about something, I do not waste much time listening to other people’s arguments (R)	0.68	0.683
	PT5 I believe that there are two sides to every question and try to look at them both	0.85	0.837
	PT6 When I’m upset at someone, I usually try to “put myself in his shoes” for a while	0.77	0.892
	PT7 Before criticizing somebody, I try to imagine how I would feel if I were in their place	0.73	0.729
**Attribution of blame and perceived responsibility** (*TLI = 0.85, CFI = 0.93, GFI = 0.89, RMSEA = 0.063, ECVI = 0.90*)			
	AB1 I think the girl should feel ashamed	0.88	0.784
	AB2 I think the girl deserves the negative consequences	0.72	0.775
	AB3 I think it is the girl’s fault for being treated this way	0.81	0.882
		0.67	0.703
	FR4 I would feel guilty as I should prevent these things from happening	0.89	0.809
	FR5 I think it is my responsibility to do something to fix the situation as soon as possible	0.71	0.793
	FR6 I think it is my responsibility to deal with this incident, even though it happened outside the school	0.86	0.693

EC means Empathetic concern, PT means Perspective taking, AB means attribution of blame, and FR mean feelings of responsibility.

### Normality and homoscedasticity test

Normality test was conducted to determine whether the data deviated from the expectation of a normal distribution. Table 3 shows both Kolmogorov–Smirnov (KS) and Shapiro–Wilk test of normality. The results were insignificant (*p* ≥ 0.05), indicating that the values were sampled from the population that follows a normal distribution. Each normality test works slightly differently and may produce different results when using the same data set (El Bouch et al., 2022). However, in this study, several tests indicated similar results of normal distribution. The results from skewness and kurtosis test also indicated values which were within an accepted range. Table 3 below shows the results in detail. Skewness and Kurtosis tests of normality are encouraged larger sample (*n* ≥ 300), and so we encourage to consider Skewness and Kurtosis test results in this study. On the other hand, the homoscedasticity test results indicated that the residuals were equally distributed.

**Table 3:** Normality test.

	Kolmogorov-Smirnov^a^			Shapiro–Wilk			Skewness		Kurtosis	
	Statistic	df	Sig.	Statistic	df	Sig.	Statistic	Std. error	Statistic	Std. error
Empathy	0.191	1,362	0.063	0.937	1,362	0.080	0.666	0.076	−1.536	0.152
Blame	0.170	1,362	0.103	0.938	1,362	0.126	1.417	0.076	1.434	0.019
Resp.	0.163	1,362	0.091	0.809	1,362	0.069	0.784	069	0.504	0.162

a Lilliefors significance correction.

### Descriptive analyses and bivariate correlations

Table 4 provides the descriptive statistics for the variables of our primary concern (empathy, attribution of blame, and feelings of responsibility) and participants’ demographics. Except for attribution of blame (M = 2.9117, SD = 0.706), the average ratings for other variables were high above the midpoint, indicating participants’ higher level of empathetic concern and feelings of responsibility toward early pregnancy among middle school girls. The bivariate correlation between the study variables supported our hypotheses (H2 and H3). There were negative correlations between participants’ attribution of blame and their empathetic concerns (*r* = −0.778) and feelings of responsibility (*r* = −0.318). In addition, the higher level of empathic concern and feelings of responsibility correlated to a lower level of attribution of blame. Participants’ age and their education level positively correlated with all variables of our primary concern (except the attribution of blame), meaning higher age and education level correlated to a lower level of attribution of blame.

**Table 4:** Correlation co-efficiencies between variables.

Variable	*M*	*SD*	1	2	3	4	5
Age	35.1741	0.996	1	-	-	-	-
Education	1.9685	0.056	0.513	1	-	-	-
Empathy	3.9390	0.873	0.764	0.394	1	-	-
Attribution of blame	2.9117	0.706	−0.631	−0.003	−0.778	1	-
Feelings of responsibility	4.1755	0.682	0.502	0.407	0.690	−318	1

### Empathic concern, attribution of blame, and feelings of responsibility between parents and teachers

Table 5 shows the group statistics between parents and teachers on empathic concern, attribution of blame, and feelings of responsibility. Parents’ mean scores of empathetic concern and feelings of responsibility were higher than teachers’ mean scores, except for attribution of blame. Teachers’ mean score of attribution of blame was higher (M Attribution of blame = 2.9548, SD Attribution of blame = 0.29688) than it was for parents (M Attribution of blame = 2.8623, SD Attribution of blame = 0.34070). The independent sample *t*-test was conducted to test the hypotheses. It helped to establish whether these mean differences (in Table 2) happened by chance in our sample or existed in the population.

**Table 5:** Group statistics on empathy, attribution of blame, and feelings of responsibility between parents and teachers.

	Gender	*N*	Mean	Std. deviation	Std. error mean
Empathy	Parents	690	4.0101	0.79518	0.02752
	Teachers	672	3.8287	0.76926	0.01653
Attribution of blame	Parents	690	2.8623	0.34070	0.01179
	Teachers	672	2.9548	0.29688	0.00638
Feelings of responsibility	Parents	690	4.1871	0.61953	0.02144
	Teachers	672	4.1429	0.63092	0.01356

### Independent sample *t*-test results on empathy, attribution of blame, and the feelings of responsibility between parents and teachers

Table 6 shows that equal variance was assumed for empathetic concern (*f* = 1.057, *p* > 0.05) and feelings of responsibility (*f* = 0.890, *p* > 0.05), but not assumed for attribution of blame (*f* = 11.644, *p* < 0.05). Supporting our hypotheses (H1 and H4), the *t*-test results indicated a significant mean difference between parents and teachers on empathy [*t*(1362) = 5.735, *p* < 0.001], attribution of blame [*t*(654.403) = 6.902, *p* < 0.001], and feelings of responsibility [*t*(1362) = 1.727, *p* < 0.001]. The average empathic concern for parents was 0.18142 higher than the average mean score for teachers, and the average attribution of blame for teachers was.09253 higher than the average for parents. In contrast, the average feelings of responsibility for parents were 0.04416 higher than the feelings of responsibility for teachers. All the results were significantly below the level of chosen significance (95% of confidence interval).

**Table 6:** *T*-test results for differences in empathy, attribution of blame, and feeling of responsibility for parents and teachers.

		Levene’s test for equality of variances		*t*-test for equality of means						
		*F*	*Sig.*	*t*	*df*	*Sig*. (2-tailed)	Mean difference	Std. error difference	95% confidence interval of the difference	
									Lower	Upper
Empathy	Equal variances assumed	1.057	0.304	5.735	516	0.000	0.18142	0.03163	0.11940	0.24345
	Equal variances not assumed			5.651	654.860	0.000	0.18142	0.03210	0.11845	0.24440
Attribution of blame	Equal variances assumed	11.644	0.001	7.334	516	0.000	0.09253	0.01262	0.06779	0.11727
	Equal variances not assumed			6.902	654.403	0.000	0.09253	0.01341	0.06623	0.11883
Feelings of responsibility	Equal variances assumed	0.890	0.345	1.727	516	0.054	0.04416	0.02557	−0.00598	0.09431
	Equal variances not assumed			1.741	654.687	0.042	0.04416	0.02537	−0.00559	0.09392

### Empathic concern, attribution of blame, and feelings of responsibility by gender (H4)

Table 7 shows the group statistics between female and male respondents on empathic concern, attribution of blame, and feelings of responsibility. The female respondents’ mean scores in empathetic concern and feelings of responsibility were higher than the males’ mean scores, except with the attribution of blame. Males’ mean score of attribution of blame was higher (M Attribution of blame = 2.9592, SD Attribution of blame = 0.29856) than it was for female (M Attribution of blame = 2.8642, SD Attribution of blame = 0.34646). The independent sample *t*-test was also conducted for testing our hypothesis (H4), and it helped to establish if these mean differences of our variables between genders happened by chance in our sample or existed in the population.

**Table 7:** Group Statistics on empathy, attribution of blame, and feelings of responsibility.

	Gender	*N*	Mean	Std. deviation	Std. error mean
Empathy	Female	728	4.0416	0.78284	0.02571
	Male	634	3.8365	0.76899	0.01632
Attribution of blame	Female	728	2.8642	0.34646	0.01138
	Male	634	2.9592	0.29856	0.00634
Feelings of responsibility	Female	728	4.2025	0.61037	0.02005
	Male	634	4.1455	0.62856	0.01334

### Independent sample *t*-test results on empathy, attribution of blame, and the feelings of responsibility

From Levene’s test (Table 8), equal variance was assumed for empathetic concern (*f* = 0.388, *p* > 0.05), and feelings of responsibility (*f* = 0.551, *p* > 0.05), but not assumed for attribution of blame (*f* = 14.554, *p* < 0.05). Supporting the hypothesis (H4), the *t*-test results showed significant mean differences between female and male respondents on empathy [*t*(1362) = 6.784, *p* < 0.001], attribution of blame [*t*(654.991) = 7.298, *p* < 0.001], and the feelings of responsibility [*t*(1362) = 2.340, *p* < 0.001]. The average empathic concern for female respondents was 0.20511 higher than the average mean score for male respondents, the average feeling of responsibility for female respondents was 0.05704 higher than the average for male respondents, while males’ mean of attribution of blame was 0.09506 higher than that of females. All the results were significantly below the chosen significance level of 95% (α = 0.05).

**Table 8:** *T*-test results for differences in empathy, attribution of blame, and feeling of responsibility by gender.

		Levene’s test for equality of variances		*t*-test for equality of means						
		*F*	*Sig.*	*t*	*df*	*Sig.* (2-tailed)	Mean difference	Std. error difference	95% confidence interval of the difference	
									Lower	Upper
Empathy	Equal variances assumed	0.388	0.533	6.784	516	0.000	0.20511	0.03023	0.14583	0.26438
	Equal variances not assumed			6.735	106.893	0.000	0.20511	0.03045	0.14537	0.26484
Attribution of blame	Equal variances assumed	14.554	0.000	7.756	516	0.000	0.09506	0.01226	0.07103	0.11909
	Equal variances not assumed			7.298	127.991	0.000	0.09506	0.01302	0.06951	0.12061
Feelings of responsibility	Equal variances assumed	0.551	0.458	2.340	516	0.019	0.05704	0.02437	0.00926	0.10483
	Equal variances not assumed			2.369	181.900	0.018	0.05704	0.02408	0.00981	0.10427

## Discussion

The general goal of this study was to assess the empathetic concern, attribution of blame, and the feelings of responsibility among teachers and parents toward early pregnancy among middle school adolescents. It is essential to know the feelings of teachers and parents on this sensitive and vital phenomenon that escalates beyond expectations, crippling youths across Tanzania and internationally. Building on the previous works (Wolak and Finkelhor, 2011b), it was predicted that parents’ and teachers’ attribution of blames and their feelings of responsibility are influenced by their level of empathy toward the victims. The findings of this study are in line with the prediction, as they indicated a higher level of empathy being correlated with a higher level of feeling of responsibility (H2) and a lower level of the attribution of blame (H3), meaning that empathetic teachers and parents feel more responsible for early pregnancy among middle school students, and rarely attribute their blame to the victims (pregnant adolescents).

Although studies have yielded inconsistent results on age issues and empathetic concern among people of different cultures (Cavallini et al., 2021; Tremblay et al., 2022), this particular study indicated the positive and significant influence of age on the empathetic concern. The evidence showed a positive and significant correlation between teachers’ teaching experience and their empathic concern, from which it can be argued that general life experience among teachers and parents positively influences the empathetic level. Although junior teachers add new energy to teaching (Nja et al., 2022), they may hardly put themselves in a victim’s shoes and perceive the problem as the victim does. Young teachers’ perspectives and feelings toward the victims of risky behaviors (i.e., early sex debut and early pregnancy) are yet to the level of their senior teachers’ counterparts.

It was also predicted based on previous literature (Pechorro et al., 2017; Tremblay et al., 2022) that females’ level of empathy, feelings of responsibility, and attribution of blame are different from that of males. Building on those past studies, the findings in this study supported the hypothesis (H6). Except for attribution of blame, female participants’ levels of empathy and their feelings of responsibility were significantly higher than that of male participants. Males’ attribution of blame was significantly lower than that of female counterparts, meaning that women may highly take responsibility for adolescent girls’ risky behaviors, while blaming the victims less. These results are very important in fighting against early pregnancy. Females (i.e., parents) spend more time with adolescents, meaning that they can play a more significant part in abolishing the problem with their more profound empathetic concern and feelings of responsibility.

Empathetic concern for children has also been discussed in the previous studies, especially toward children of different races in multiracial countries like the United States (Wang et al., 2020). However, this study has added new insights from one of the developing countries by comparing empathic concerns between parents and teachers for pregnancy among middle school students. A significant difference in empathy levels between parents and teachers toward pregnancy problems has been indicated, which is also reflected in their attributing blame to the victims. Although theoretical studies may suggest that parents’ empathy toward any child is natural, a pretty different picture is printed from our findings. In line with a few literature (Young et al., 2017), the findings from this study have revealed that teachers (including females) feel being concerned about students’ behaviors outside school would mean overstepping the school authority. Sometimes the school may even blame the victims of risky behavior or early pregnancy (Jørgensen et al., 2019).

However, all participants’ general feelings of responsibility for early pregnancy are beyond the midpoint, indicating that they all feel responsible for the problem. Nevertheless, it can further be argued that because the feelings operate under different perspectives and feelings (empathy), their reaction to the problem can also be of varying intensity. Being in adolescents’ shoes (empathizing) and taking their perspectives may help teachers and parents understand their difficulties and finally come up with solutions (Liew and Fadil Azim, 2022). The findings reveal that higher feelings of responsibility relate to lower attribution of blame among teachers and parents, meaning that most teachers with deeper empathetic concern tend to blame the targets less. Aligning to few previous studies (Schneider and Arnot, 2018; Bordalba and Bochaca, 2019), teachers, parents, and students need to work together and take responsibility for the problem.

The higher parents’ feelings of responsibility mean they perceive higher responsibility for early pregnancy among adolescents than teachers. However, the higher level of education attained correlates with higher feelings of responsibility for both parents and teachers. These results indicate the importance of education and its role in effectively fulfilling a child-rearing obligation. Children from educated parents have less probability of engaging in risky behavior (Liu and Yang, 2022). The findings in this study add to the existing literature by highlighting parents’ level of education, empathy, blame attribution, and feelings of responsibility. In addition, looking at pregnant adolescents as responsible for their victimization (attribution of blame) correlated with lower empathy and feelings of responsibility.

### Strengths and limitations

In this study, the criteria which were used to split teachers and parents into two different groups are: (1) responsibility differences, (2) time spent with adolescent students per day, and (3) attachment and connection. However, teachers are also parents to their children. Their feelings of responsibility, empathy, and attribution of blame may also be influenced by their being parents as well. In the future study, parents whose adolescents have experienced early pregnancy can be treated as a group to compare with those parents who have never experienced their children getting pregnant during their adolescent period. There are parents who are very occupied with their jobs, and for that, they do not spend a long time with their children, meaning that in this context, teachers may form a stronger attachment with children than their parents do.

Students are also responsible for risky behaviors (i.e., early sex debut and unprotected sex,) which result in early pregnancy and infection with sexually transmitted diseases (STDs). However, in this study, the feelings of responsibility and attribution of blame were not measured from them. Future studies can also explore students’ feelings of responsibility toward their pregnancy. In this way, the intervention programs may be effective, especially after knowing the level of understanding among adolescents about their responsibility and self-blame (guilt) for their pregnancy.

### Practical implications

The prevention and intervention program for early pregnancy among middle school students needs to create awareness among parents and teachers on their responsibilities. Teachers and parents can be essential in preventing risky behaviors, including early sex debut, unprotected sex, and sharing sexual images (sexting). Because early pregnancy affects adolescents’ education and school lives, it is also imperative for teachers to feel responsible for the intervention. Although the previous studies have indicated that teachers feel responsible for educating students about delaying sex (postponing), safe sex, and their advantages like avoidance of legal consequences (Hayes et al., 2013), special training on how to deal with the problem of early pregnancy would benefit many teachers who hesitate to do so.

Our findings have also demonstrated the influence of individual factors like empathy on perceiving the problem of early pregnancy among adolescents. Pre-service teacher education programs and in-service training should encourage empathy and consider empathy as a critical element of being a teacher. Teachers’ and parents’ empathy toward the victims of early pregnancy increases the likelihood of engaging with the intervention programs and supporting the targets. Future programs may also involve training parents because adolescents spend more time with their parents than teachers. In addition, because early pregnancy affects society in general, everyone needs to take responsibility for abolishing all the risky behaviors that may result in early pregnancy and school dropout.

## Conclusion

Teenage pregnancy is a public health problem because of its considerable personal, social, and medical impacts on adolescents and the general population. The responsibility for preventing adolescent pregnancy lies in the hands of everyone, including adolescents themselves. Preventive measures and programs implemented under different policies continue to work, especially in developing countries. However, understanding the social feelings about their responsibilities is very important. To successfully combat early pregnancy and its associated impacts (i.e., deaths), every social member must assume responsibility and avoid the blame game. Higher attribution of blame, as it stands with the findings of this study, correlates with lower feelings of responsibility. Although women are known for their empathetic character toward the victims, teacher training programs need to integrate empathy with both men and women because teaching needs perspective-taking and empathy arousal. Teachers are parents need to work together toward preventing adolescents’ risk behaviors like early sex debut, unprotected sex, and finally, unplanned pregnancy.

This study acts as an alarm toward teachers’ in-service training and the content into which the emphasis is put. The responsibility of ensuring students’ safety, both physically and psychologically, lies on the hand of teachers and parents. Both parents and teachers need feel responsible for early pregnancies among girl students, empathize with students, and find the solution toward the problem. The question remains on ways through which teachers empathize more than they do, feeling more responsible and reducing blaming. Because the problem of early pregnancy is becoming common throughout developing countries, teachers’ colleges and all the training institutions need to consider extensiveness of training content. The results from this study are very important for parents, teachers, students, and government at large. Everyone is responsible toward fighting against early pregnancy among students, including the victims. Attributing blames to the victims reduces empathetic concern and the feelings of responsibility among social members.

## Data availability statement

The original contributions presented in the study are included in the article/supplementary material, further inquiries can be directed to the corresponding author.

## Ethics statement

The studies involving human participants were reviewed and approved by the Ethics Committee of Zhejiang Normal University’s College of Teacher Education. The patients/participants provided their written informed consent to participate in this study.

## Author contributions

AF and BS designed the study, collected data from students, analyzed the data by using SPSS, wrote the first draft of the manuscript, worked on ethical approval, and collected all the information from the college. MO codded the data, prepared all the figures, proofread, and prepared the last version of the manuscript. All authors contributed to the article and approved the submitted version.

## Funding

This work was supported by the National Natural Science Foundation of China (grant number 31871124).

## Conflict of interest

The authors declare that the research was conducted in the absence of any commercial or financial relationships that could be construed as a potential conflict of interest.

## Publisher’s note

All claims expressed in this article are solely those of the authors and do not necessarily represent those of their affiliated organizations, or those of the publisher, the editors and the reviewers. Any product that may be evaluated in this article, or claim that may be made by its manufacturer, is not guaranteed or endorsed by the publisher.

## Appendix

**Appendix A: Exposure to the broader reality of early pregnancy and its associated effects.**

Globally, 15% of adolescent girls give birth before they turn 18. At least 21 million adolescent girls 15–19 years old get pregnant annually in developing countries, and 48% of them are unintended. At least 5.6 million abortions occur annually, and 70% are unsafe, leading to severe problems like morbidity, maternal mortality, and lifetime health problems. Maternal conditions are in the top five causes of death among adolescents (15–19 years), and developing countries contribute 99% of them. Early pregnancies may also lead to issues of rejection, stigmatization, and forced early marriage. Tanzania specifically has 3.6 million primary and secondary-aged girls out of school because of pregnancy, and 5,500 continue to drop out annually.

**Appendix B: Interpersonal Reactivity Index (IRI) items by Davis (1983).**

*Empathic concern (EC)*

1. I often have tender, concerned feelings for people less fortunate than me
2. Sometimes I do not feel very sorry for others when they have problems. (Reversed)
3. When I see someone being taken advantage of, I feel protective toward them.
4. Other people’s misfortunes do not usually disturb me a great deal. (Reversed)
5. When I see someone being treated unfairly, I sometimes do not feel very much pity for them. (Reversed)
6. I would describe myself as a pretty soft-hearted person.

*Perspective-taking (PT sub-scale)*

1. I sometimes find it difficult to see things from the “other guy’s” point of view. (Reversed)
2. I try to look at everybody’s side of a disagreement before making a decision.
3. I sometimes try to understand my friends better by imagining how things look from their perspective.
4. If I’m sure I’m right about something, I do not waste much time listening to other people’s arguments (Reversed)
5. I believe that there are two sides to every question and try to look at them both.
6. When I’m upset at someone, I usually try to “put myself in his shoes” for a while.
7. Before criticizing somebody, I try to imagine how I would feel if I were in their place.

**Appendix C: Attribution of blame and perceived responsibility items by Sciacca et al. (2021).**

1. I think the girl should feel ashamed (attribution of blame).
2. I think the girl deserves the negative consequences (attribution of blame).
3. I think it is the girl’s fault for being treated this way (attribution of blame).
4. I would feel guilty as I should prevent these things from happening (perceived responsibility).
5. I think it is my responsibility to do something to fix the situation as soon as possible (perceived responsibility).
6. I think it is my responsibility to deal with this incident, even though it happened outside the school (perceived responsibility).
